# Programming the Brain: How Maternal Overnutrition Shapes Cognitive Aging in Offspring

**DOI:** 10.3390/nu17060988

**Published:** 2025-03-12

**Authors:** Pratheba Kandasamey, Daria Peleg-Raibstein

**Affiliations:** 1 Institute for Neuroscience, Institute of Food Nutrition and Health, Department of Health Sciences and Technology, ETH Zurich, Schorenstrasse 16, 8603 Schwerzenbach, Switzerland; pratheba.kandasamey@hest.ethz.ch; 2 Neuroscience Center Zurich, University of Zurich/ETH Zurich, 8057 Zurich, Switzerland

**Keywords:** maternal, overnutrition, mice, cognition, aging, memory, offspring

## Abstract

**Background:** Maternal overnutrition critically influences offspring’s long-term metabolic and cognitive health. While prior research indicates maternal diet can disrupt hippocampal function, the specific impact on spatial memory remains unclear. **Methods:** Female mice were fed a high-fat diet (HFD) for nine weeks before and during pregnancy. Offspring were weaned onto a standard diet and tested at postnatal day 90 using the dry maze, a spatial reference memory task. **Results:** HFD-exposed offspring exhibited significant learning acquisition impairments, with prolonged latencies in locating hidden rewards and diminished within-session improvements compared to controls. During the probe trial, they spent significantly less time in the target quadrant, indicating long-term spatial memory retention deficits. Notably, these cognitive impairments occurred independently of body weight differences at testing. **Discussion:** This study uniquely demonstrates that maternal HFD exposure induces specific spatial memory deficits in adult offspring, potentially through neurodevelopmental alterations preceding metabolic dysfunction. The results highlight the importance of prenatal nutrition in shaping cognitive outcomes later in life. **Conclusions:** These findings extend our understanding of how prenatal nutrition impacts cognitive aging and disease susceptibility. Given rising obesity rates among women of reproductive age, this research underscores the urgent need for targeted interventions to mitigate the intergenerational effects of maternal overnutrition on brain function.

## 1. Introduction

The global prevalence of obesity has reached alarming levels, posing significant challenges to healthcare systems worldwide. As of 2022, an estimated 2.5 billion adults worldwide were classified as overweight, including approximately 890 million individuals living with obesity. This indicates that 43% of adults were overweight, and 16% were living with obesity [1]. Obesity is linked to a higher prevalence of comorbid conditions, including dyslipidemia, hypertension, and type 2 diabetes mellitus (T2DM) [2]. A growing concern is the increasing rate of obesity among women of reproductive age. Recent data indicate that nearly two thirds of women aged 19 to 44 are overweight, and 36.5% are obese. Consequently, this epidemic extends to younger populations, with nearly 37 million children under the age of five classified as overweight in 2022 [1]. The etiology of this obesity epidemic extends beyond factors such as an affluent lifestyle, reduced physical activity, or genetic predisposition. Emerging evidence suggests that environmental influences during fetal development play a crucial role. The “Developmental Origins of Health and Disease” (DOHaD) hypothesis posits that variations in maternal nutrition can have profound and long-term implications for the health of offspring, such as obesity, metabolic syndrome, and cardiovascular disease [3,4]. Programming refers to the concept that exposure to certain factors during key developmental periods can have lasting effects. The Barker hypothesis suggests that alterations in maternal nutrient supply to the fetus can significantly impact the long-term health of the offspring [for review see [5]].

While initial research focused on the effects of fetal undernutrition, recent studies have shifted attention to maternal overnutrition [6]. Epidemiological data indicate a positive relationship between birth weight and adult body mass index (BMI), with maternal BMI and gestational weight gain being significant predictors of offspring obesity [7]. A U.S. cohort study reported that maternal obesity during the first trimester was associated with a two- to threefold higher likelihood of childhood obesity. By the age of four, 24% of children born to obese mothers were classified as obese, compared to 9% of those born to mothers with a normal weight [8].

Beyond metabolic outcomes, maternal obesity has been linked to cognitive impairments in offspring [for review see [9]]. Studies have reported associations between maternal obesity and reduced IQ scores [10], lower cognitive test performance [11], and increased risk of intellectual disabilities in children [12]. Additionally, maternal obesity has been associated with a higher incidence of attention-deficit/hyperactivity disorder (ADHD) symptoms in offspring [13,14,15].

Although the association between maternal obesity and offspring’s cognitive disabilities was described, the current literature in humans is still scarce and findings are inconsistent [9]. The underlying mechanisms linking maternal overnutrition to cognitive abnormalities in offspring remain under investigation. It is hypothesized that maternal obesity may alter the in utero environment, affecting fetal brain development and increasing susceptibility to cognitive impairments later in life. Cognitive impairments observed in adult offspring may also stem from obesity itself. Research indicates that excessive caloric intake and obesity are linked to a heightened risk of cognitive deficits and various forms of neurodegenerative dementia [16,17]. However, it remains unclear whether cognitive decline is a predisposing factor for obesity or if the metabolic state directly contributes to cognitive dysfunction. Another key question is whether maternal overnutrition accelerates metabolic and cognitive aging across generations. Animal models have been instrumental in elucidating these mechanisms, providing insights that are challenging to obtain from human studies due to ethical and practical constraints. Given these associations, there is a growing need for public health strategies aimed at improving maternal health before and during pregnancy. Nutritional interventions, such as optimizing preconception and prenatal diets, promoting healthy gestational weight gain, and ensuring adequate micronutrient intake, may play a crucial role in mitigating the risk of cognitive and metabolic impairments in offspring. Several initiatives, such as the WHO’s maternal nutrition guidelines [18] and targeted obesity prevention programs, highlight the importance of early nutritional interventions in shaping long-term health outcomes.

Given the rising rates of global obesity, understanding the impact of maternal overnutrition on both metabolic and cognitive outcomes in subsequent generations is imperative. This knowledge is crucial for developing effective interventions to mitigate the long-term health consequences for offspring. Integrating findings from studies on maternal overnutrition into public health frameworks can inform policies aimed at reducing childhood obesity and cognitive impairments. Public health efforts should focus on early-life interventions, including maternal dietary guidance, community-based health programs, and policies to improve food quality and accessibility for expectant mothers. Strengthening these preventive measures can have profound implications for future generations, ultimately reducing the burden of metabolic and neurodevelopmental disorders globally.

## 2. Materials and Methods

### 2.1. Animals and Housing

Female and male C57BL/6 breeders were obtained from Charles River Laboratories (Charles River, Erkrath, Germany). Mice were housed under constant temperature and humidity conditions (21 ± 1 °C and 55 ± 5%, respectively) with a 12:12 h light–dark cycle (lights off at 7.00 a.m.). Animals had ad libitum access to standard rodent chow (Kliba 3430, Klibamühlen, Kaiseraugst, Switzerland) and water. The procedure described in the present study was in compliance with the Veterinary Office of the Canton of Zurich and in agreement with the Swiss regulations on animal experimentation.

### 2.2. Maternal High-Fat Diet Exposure

Female mice were fed a high-fat diet (HFD) (Provimi Kliba, Kaiseraugst, Switzerland; providing 60% of energy from fat) for a total of nine weeks—starting three weeks before mating, continuing through gestation, and extending into the three-week lactation period. Control dams were maintained on standard rodent chow, as previously described [19]. Both control and HFD offspring had ad libitum access to standard rodent chow from weaning (postnatal day 21). The animals were randomly allocated from 8 to 10 litters: HFD and control offspring [N = 16 (8 males, 8 females)]. Mice were left undisturbed in the animal facility until behavioral testing commenced on postnatal day 90.

### 2.3. Reference Memory Tested in the Dry Maze

In this study, we selected the dry maze task over the Morris water maze (MWM) to assess cognitive impairments in mice. This decision was influenced by evidence suggesting that mice may experience greater difficulty with swimming tasks compared to rats. Research indicates that while mice perform comparably to rats in dry-land mazes, they exhibit impairments in place and matching-to-place learning when tested in swimming pools [20]. These findings suggest that the MWM may introduce confounding variables related to swimming ability in mice, potentially affecting the assessment of cognitive functions. Additionally, it eliminates potential motor function confounds, ensuring that performance differences are attributed to cognitive deficits rather than physical limitations.

**Figure 1:**
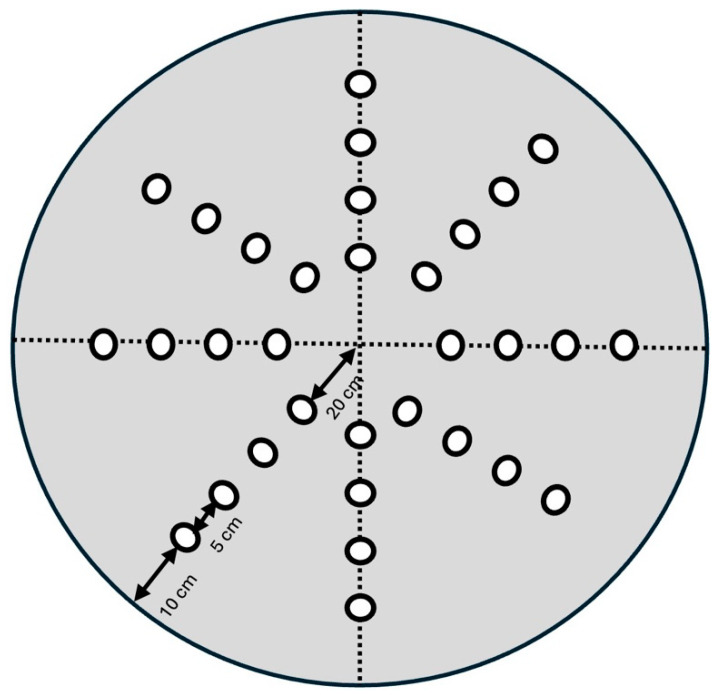
Diagram of the dry maze, a test arena made from a wooden circular board (1.1 m in diameter) which was painted gray and designed for reference memory assessment. It featured 32 symmetrically arranged wells (3.1 cm in diameter, 1.3 cm deep) to prevent mice from relying on local cues to locate the target. The wells were positioned radially, spanning distances of 20 to 45 cm from the center, resembling the layout of a radial arm maze. Each well was fitted with a plastic bottle cap, and the reward consisted of 75 μL of freshly prepared diluted condensed milk. Dashed lines indicated one of four possible quadrant configurations of the board.

The test arena was made from a wooden circular gray painted board as fully described previously [21] (See Figure 1). The board’s surface featured 32 wells arranged in a radial pattern, each capable of holding either a visual cue mounted on a 15 cm stick or a reward (75 μL of freshly prepared condensed milk). To assess performance in each trial, an automated EthoVision tracking system (Noldus Information Technology, Wageningen, the Netherlands) was used to record the time taken and the distance traveled by the animal to locate the reward.

For the purpose of the test the animals were gradually food-deprived during the initial habituation phase (days 1–5). During this period the animals experienced 2 daily trials on the dry maze for 2 min each. During this stage no reward was presented. Pretraining phase (days 6–7): animals were pretrained in the dry maze with a visual cue placed near the rewarded well for two consecutive trials. Each animal was left on the maze for 2 min; however, if the animal failed to reach the reward it was guided by the experimenter. Reference memory test (days 8–11): each animal was subjected to 4 daily trials with an inter-trial interval (ITI) of 2 min. The reward was placed in the same well throughout all trials across the 4 testing days. Similar to the pretraining phase, animals were allowed a maximum of 2 min to locate the reward. If the animal did not locate the reward the experimenter gently guided the animal to the baited well. Learning acquisition was measured by the decrease in latency or distance traveled to locate the reward over consecutive test days and trials. On the probe test (day 12), animals were given 2 min to explore the dry maze without the presence of a reward.

### 2.4. Statistical Analysis

Data analysis was conducted using IBM SPSS Statistics (version 26). The results are presented as mean ± standard error, as summarized by SPSS. Where applicable, repeated measures factors such as days, trials, delays, and quadrants were incorporated into the analysis. Significant main effects or interactions were further examined using Fisher’s least significant difference (LSD) post hoc test. A significance threshold of *p* < 0.05 was applied to all statistical analyses.

## 3. Results

### 3.1. Body Weight

A 2 × 2 ANOVA was conducted on the body weight of offspring post-weaning, categorized by sex and exposure to maternal HFD. The analysis revealed a significant main effect of sex on body weight (*F*_1,20_ = 22.070, *p* = 0.0001), indicating that males and females significantly differed in their body weight. However, the main effect of treatment was not significant, suggesting that exposure to a maternal HFD did not significantly affect body weight when compared to controls.

### 3.2. Spatial Reference Memory in the Dry Maze

#### 3.2.1. Visual Cue Task (Pretraining)

**Figure 2:**
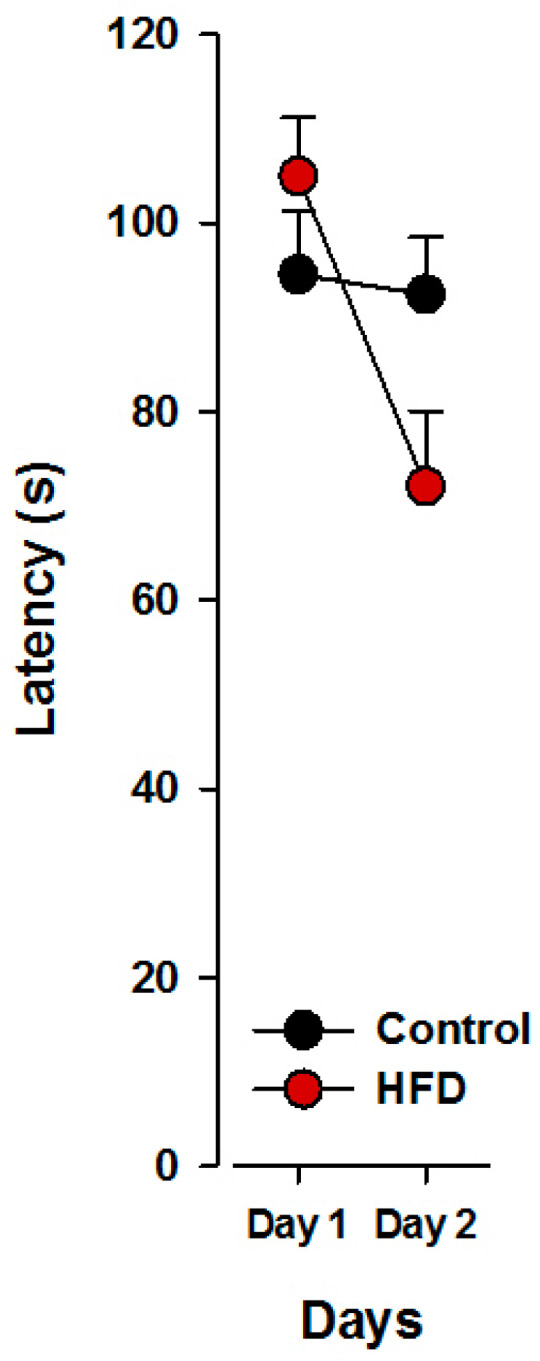
The latency to find the rewarded well was reduced from day 1 to day 2 during the pretraining phase in the HFD offspring compared to control offspring. N(Control) = 12 (6 m, 6 f), N(HFD) = 12 (6 m, 6 f). All values are means ± SEM. f, females; m, males.

An overall reduction in the latency to obtain the reward from day 1 to 2 was detected in the visual cue part of the task. Offspring born to HFD mothers displayed a reduction in latency from day 1 relative to day 2, whilst offspring born to control mothers showed an attenuated improvement. These observations were supported by a 2 × 2 × 2 × 2 (maternal exposure × sex × day × trials) ANOVA of latency yielding a significant main effect of days (*F*_1,20_ = 7.34; *p* < 0.02) and a significant interaction of maternal exposure x days (*F*_1,20_ = 5.70; *p* < 0.03; Figure 2). Post hoc analysis further confirmed a significant day effect in HFD offspring, with a reduction in latency on day 2 compared to day 1 (*p* < 0.05), indicating a quicker reach to the rewarded well on the second training day. Neither the main effect of sex nor its interaction with maternal exposure were significant.

#### 3.2.2. Reference Memory

**Figure 3:**
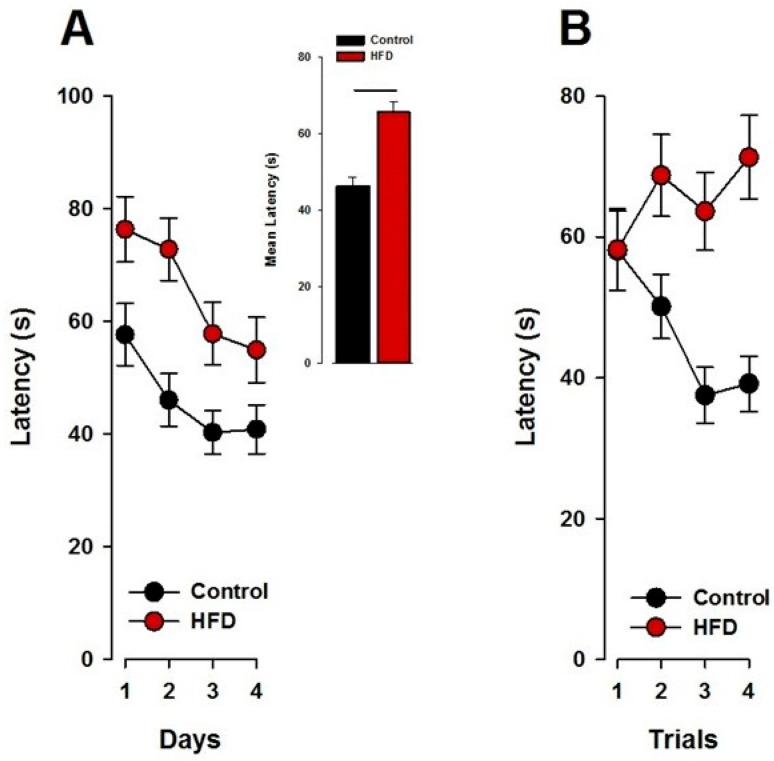
(**A**) The latency to reach the rewarded well during the 4 days of acquisition indicates that HFD offspring showed a slower performance curve as compared to control offspring. The incent bar plot on the right depicts the mean performance expressed as the percentage of correct responses during the acquisition phase. (**B**) Offspring born to HFD mothers did not improve their performance between trials while the latency of the control offspring was reduced between trials. N(Control) = 12 (6 m, 6 f), N(HFD) = 12 (6 m, 6 f). All values are means ± SEM. f, females; m, males.

Learning was indexed by a shortening of the time (latency) to find the hidden reward over days and trials within days. Performance showed a progressive improvement across test days, as reflected by a significant main effect of days (*F*_3,60_ = 8.32; *p* < 0.001). Offspring born to control and HFD mothers displayed a clear reduction in the latency to find the rewarded hole. However, the HFD offspring started from higher latency levels and at the end of training did not reach as low latency levels as the control offspring (Figure 3A). Offspring born to control mothers showed a significant reduction from trials 1 to 4, whilst offspring born to HFD-exposed mothers failed to show any improvement within trials. These observations were supported by a 2 × 2 × 4 × 4 (maternal exposure × sex × days × trials) ANOVA of latency, which revealed a main effect of maternal exposure (*F*_1,20_ = 6.07; *p* < 0.03; Figure 3A) and a significant interaction of maternal exposure x trials (*F*_3,60_ = 5.48; *p* < 0.003; Figure 3B).

#### 3.2.3. The Probe Test

**Figure 4:**
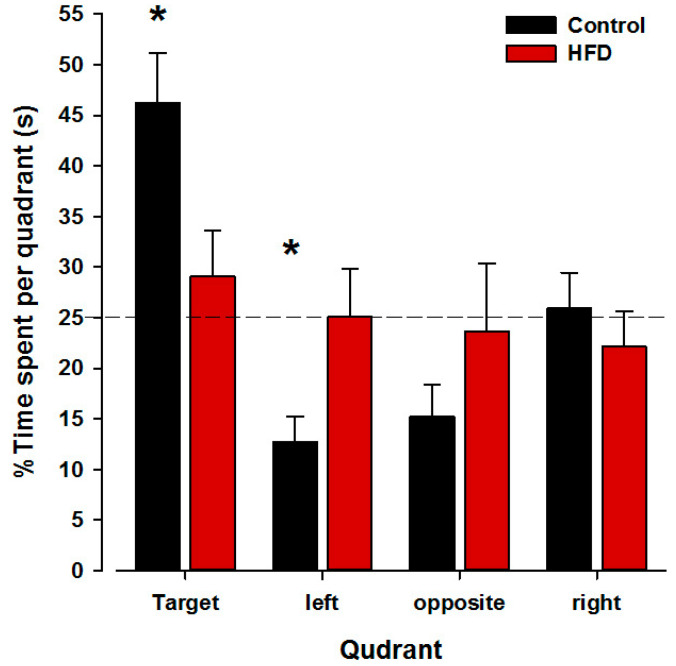
Percentage of time spent in each quadrant during the probe trial. Control offspring demonstrated strong retention of the previously rewarded well, whereas HFD offspring exhibited impairments in this task. The dashed line represents chance level, and asterisks denote a significant preference for the indicated quadrant over the others, based on significant pairwise comparisons (*p* < 0.05). N(Control) = 12 (6 m, 6 f), N(HFD) = 12 (6 m, 6 f). All values are means ± SEM. f, females; m, males.

On the probe trial, there was a significant quadrant effect (*F*_3,66_ = 5.95; *p* < 0.002) and a significant quadrant × group effect (*F*_3,66_ = 3.45; *p* < 0.03; Figure 4). Subsequent post hoc analysis comparing the target-quadrant exploration within groups revealed a significant quadrant effect in HFD offspring (*p* < 0.02) for the target quadrant and in the left quadrant (*p* < 0.04). HFD offspring spent significantly less time in the target quadrant as control offspring and spent respectively more time in the left quadrant.

## 4. Discussion

This study shows that maternal overnutrition over a nine-week period leads to deficits in learning and memory, as evaluated through a spatial reference memory task using the dry maze paradigm. These findings align with previous research indicating that maternal high-fat diet (HFD) exposure results in cognitive deficits that emerge during adulthood. Specifically, our earlier studies have shown that offspring exposed to maternal HFD display impairments in reference memory tasks, such as the T-maze, while exhibiting intact working memory in Y-maze tasks across various delays [22]. Interestingly, when tested at 18 months, these animals demonstrated significant impairments in both working memory and reference memory, with performance declines in the Y-maze and T-maze tasks, respectively. Learning deficits in the two-way active avoidance paradigm further support the notion of age-dependent cognitive decline in HFD offspring, independent of locomotor activity differences [22].

By assessing maternal HFD-exposed offspring across different developmental time points, we identified specific windows of vulnerability to memory deficits with aging. Notably, extending retention times in the Y-maze revealed long-term memory impairments, highlighting the enduring effects of maternal HFD exposure on cognitive function [22]. In addition to cognitive impairments, we observed an age-dependent obesity phenotype in HFD offspring, characterized by increased body weight, altered plasma metabolic markers, and elevated fat composition [22,23]. Given that adult obesity is linked to cognitive dysfunction and heightened risk for neurodegenerative disorders such as Alzheimer’s disease [24], it is plausible that metabolic alterations contribute to cognitive impairments in HFD offspring. However, our data suggest that the observed memory deficits may not be directly attributable to metabolic dysfunction, as metabolic alterations were not evident until postnatal day 120 [23], whereas cognitive testing occurred at postnatal day 90, when body weight differences were not yet significant. The implications of these findings extend beyond animal models, as evidence from human studies suggests that maternal obesity and poor nutrition during pregnancy can negatively impact offspring’s cognitive function. Studies indicate that children born to mothers with high pre-pregnancy BMI exhibit lower performance on neurodevelopmental tests, including measures of executive function, attention, and verbal skills [25,26]. Moreover, prenatal exposure to maternal obesity has been linked to an increased risk of neurodevelopmental disorders such as attention-deficit/hyperactivity disorder (ADHD) and autism spectrum disorder (ASD) [9,14,15,27]. These findings suggest that optimizing maternal nutrition before and during pregnancy may serve as a preventive strategy to mitigate the risk of long-term cognitive deficits in offspring. In particular, studies have shown that maternal dietary improvements, such as increasing omega-3 fatty acid intake, optimizing micronutrient levels (e.g., folate, iron, and choline), and reducing high-sugar and high-fat dietary patterns, can improve offspring neurodevelopmental outcomes [28,29]. Ensuring adequate maternal intake of essential nutrients during pregnancy could help support fetal brain development, reducing the likelihood of cognitive impairments and associated neuropsychiatric conditions. Given the rising prevalence of maternal obesity and metabolic disorders, further human trials are needed to evaluate the long-term benefits of maternal dietary interventions in improving offspring cognitive health.

Recent studies have provided further insight into the mechanisms by which maternal HFD affects offspring brain development and cognitive function, which may underlie the cognitive impairments observed in our study. Bordeleau and colleagues demonstrated that maternal HFD in mice induces cerebrovascular and microglial alterations [30,31], which can impact neural connectivity and synaptic function, potentially leading to cognitive deficits in learning and memory. Additionally, Gawlińska et al. reviewed evidence from both rat and mouse models, emphasizing that maternal diet alters neurodevelopmental trajectories, contributing to impaired spatial learning and memory retention [32]. Their findings suggest that maternal HFD may disrupt key neurodevelopmental processes, including synaptic plasticity and neurotransmitter balance. Furthermore, Hawkes et al. [33] identified significant alterations in the neurovascular unit of adult offspring following maternal HFD exposure, with disruptions observed in key components such as apolipoprotein E and fibronectin, which are known to affect blood flow and nutrient delivery to the brain. Such vascular impairments could contribute to the reduced cognitive performance we observed, as proper cerebrovascular function is crucial for learning and memory. These studies collectively support the hypothesis that maternal HFD impacts cognitive function in offspring through a combination of neuroinflammatory, vascular, and synaptic alterations [34], which align with the cognitive impairments detected in our study.

While maternal HFD exposure is linked to cognitive deficits, the underlying neurobiological mechanisms remain to be fully elucidated. One key factor is synaptic plasticity, as maternal HFD has been shown to reduce long-term potentiation (LTP), decrease dendritic spine density, and lower synaptic protein expression (e.g., synapsin-1, PSD-95), impairing hippocampal and prefrontal cortex function [35,36]. Additionally, neurogenesis in the dentate gyrus (DG) appears disrupted, with reduced doublecortin (DCX) and Ki67 expression, markers of immature neurons and proliferation, respectively [37,38,39,40]. Neuroinflammation may further contribute to these impairments, as elevated pro-inflammatory cytokines (IL-6, TNF-α, IL-1β) in the hippocampus and PFC of HFD offspring suggest a neuroimmune component to cognitive dysfunction [41,42]. Additionally, epigenetic modifications, such as increased DNA methylation of brain-derived neurotrophic factor (BDNF) promoters, have been reported, potentially reducing BDNF expression and impairing synaptic function [43,44]. These findings suggest that maternal HFD influences multiple neurodevelopmental pathways, and future studies should explore whether targeting synaptic plasticity, enhancing neurogenesis, reducing inflammation, or reversing epigenetic modifications could mitigate these deficits.

Previous research has demonstrated that maternal HFD exposure induces hippocampal and prefrontal cortex alterations. For example, maternal HFD influences amino acid levels in these brain regions, which may underlie the observed cognitive deficits [45]. Additionally, reductions in hippocampal brain-derived neurotrophic factor (BDNF) levels were reported to correlate with spatial memory impairments [43,46]. These neurobiological changes may underlie the long-lasting cognitive deficits observed in HFD offspring. While the hippocampus is central to spatial memory, maternal HFD may also affect other regions, such as the prefrontal cortex (PFC) and amygdala, which contribute to cognitive flexibility, emotional regulation, and learning strategies [22,47,48,49]. PFC dysfunction in HFD offspring has been linked to impaired executive function and altered dopamine signaling, potentially influencing task performance [49,50]. Additionally, dysregulation of the hypothalamic–pituitary–adrenal (HPA) axis may contribute to the observed cognitive impairments. Studies show that HFD-exposed offspring exhibit elevated corticosterone levels, heightened stress reactivity, and increased anxiety-like behaviors, all of which can interfere with learning and memory [19,51,52,53]. Amygdala hyperactivity and increased glucocorticoid receptor expression in stress-related brain regions may further contribute to altered exploratory behavior, reducing engagement in cognitive tasks [54,55].

Another key factor influencing offspring cognitive development is placental function, which regulates fetal nutrient supply and metabolic programming. Maternal HFD has been shown to impair placental nutrient transport, increase oxidative stress, and induce inflammation, leading to altered fetal brain development [42,56,57,58,59]. Changes in placental lipid metabolism and insulin signaling have been implicated in fetal programming of metabolic and neurodevelopmental disorders, which may contribute to cognitive impairments in offspring [60,61]. Beyond gestational factors, maternal care behavior has also been considered as a possible contributor to cognitive outcomes in HFD offspring. However, in our previous study, we directly measured maternal behavior in our MHFD model and found no significant differences between HFD-exposed and control dams [23]. However, other studies have reported that maternal HFD exposure can alter maternal–infant interactions, including reduced licking and grooming behaviors, which are known to influence offspring neurodevelopment [62]. These discrepancies may be attributed to differences in HFD composition, strain-specific effects, or environmental factors, warranting further investigation. Overall, our findings may support the idea that intrauterine factors, such as disrupted placental function and fetal metabolic reprogramming, play a key role in shaping offspring neurodevelopment.

Our findings also indicate that maternal HFD exposure influences cognitive function across multiple generations. Second- (F2) and third-generation (F3) offspring, despite never being directly exposed to HFD, exhibited increased prepulse inhibition (PPI) and impaired learning in the two-way active avoidance task. These deficits were accompanied by alterations in excitatory and inhibitory amino acid levels in the prefrontal cortex and hippocampus, suggesting a transgenerational impact of maternal diet on neurodevelopment [45]. Notably, both F2 and F3 offspring displayed an obesogenic phenotype, further reinforcing the heritable metabolic consequences of maternal overnutrition [45].

Evidence from human and animal studies suggests that normal aging is associated with cognitive decline, including deficits in working memory and spatial learning [63,64]. These deficits are well documented in spatial reference and working memory tasks, particularly following hippocampal lesions [65,66]. Memory impairments in aged individuals are associated with hippocampal circuit alterations and synaptic plasticity deficits [63,64,67,68]. Our findings demonstrate that maternal HFD exposure leads to impairments not only during the acquisition phase but also in retention tasks, aligning with the well-established age-related decline in memory function [69]. While acquisition rates decline with age, memory retention is typically preserved in cognitively healthy individuals [70]. Given the detrimental impact of maternal HFD on offspring cognitive function, an important question arises: *can these deficits be ameliorated through early interventions?* Emerging evidence suggests that cognitive training and nutritional supplementation may offer promising avenues for mitigating these effects. Cognitive enrichment strategies, such as environmental enrichment and memory training, have been shown to enhance synaptic plasticity and improve cognitive outcomes in animal models of neurodevelopmental impairment [71]. Additionally, early-life interventions, including omega-3 fatty acid supplementation, have been reported to restore hippocampal function and improve memory performance in offspring of HFD-fed dams [72,73]. Another promising candidate is the antioxidant N-acetyl-cysteine (NAC), which has been identified as a potential therapeutic agent for neuropsychiatric disorders [74]. NAC supplementation during fetal life has been shown to improve glucose tolerance in offspring exposed to maternal HFD [74]. Moreover, supplementation with methyl donors has also been investigated as a potential intervention to counteract the effects of maternal HFD exposure [75].

These findings suggest that targeted interventions may help counteract the adverse neurodevelopmental effects of maternal overnutrition. Importantly, the negative consequences of maternal obesity exposure may manifest at different time points throughout life [76], from early development to adolescence and even into aging. This raises the possibility that different windows of intervention exist, with earlier interventions potentially yielding greater and longer-lasting benefits. Ideally, interventions should be implemented during critical periods of brain development, before these windows close, and likely before the end of adolescence, when neurodevelopmental plasticity is still high. Further research is required to determine the optimal timing, duration, and efficacy of such interventions in preventing or reversing cognitive impairments associated with maternal obesity.

The probe trial serves as a critical measure of hippocampally dependent spatial navigation and reference memory [77]. Our results demonstrate that control offspring spent significantly more time in the target quadrant compared to HFD offspring, as shown in Figure 4. This suggests that HFD-exposed offspring have a diminished ability to recall the learned platform location, indicating reference memory deficits. Additionally, the significant difference observed in the left quadrant further supports altered spatial navigation strategies in HFD offspring. Deficits in probe trial performance suggest disruptions in learning rather than performance, as this measure is insensitive to variations in swimming speed [78]. Furthermore, administering the probe trial 24 h after the last acquisition day allows differentiation between short- and long-term memory, which is essential for evaluating reference memory independently of the last training session [79].

In agreement with prior research, we observed age-related deficits in working memory performance, with aged HFD offspring displaying significant impairments in the Y-maze task at longer delays compared to age-matched controls [80]. These findings are consistent with studies demonstrating an age-related decline in spatial learning, including deficits in the Morris water maze [81]. Notably, aged rats typically require more time to learn the platform location but perform comparably in cued trials, indicating selective deficits in spatial learning rather than general task performance. Hippocampal synaptic alterations likely contribute to the observed cognitive deficits. Reductions in N-methyl-D-aspartate (NMDA) receptor expression, particularly NR2B subunit levels, have been implicated in age-related cognitive decline [82]. Moreover, prolonged exposure to stress is recognized as a factor that exacerbates cognitive decline and increases the risk of neuropsychiatric disorders [83]. Chronic stress has been shown to impair hippocampal-dependent learning in various spatial tasks, including the radial-arm maze, Y-maze, and Morris water maze [84]. Given that maternal obesity is associated with increased prenatal stress and neuroinflammation, these factors may further contribute to cognitive deficits in HFD offspring.

Interestingly, during the visual cue task in the pretraining stage, offspring of maternal HFD exposure exhibited a significant reduction in latency to obtain the reward from day 1 to day 2 compared to control offspring. This was the only stage in which maternal HFD-exposed offspring outperformed their controls. Previous research, including our own, has demonstrated that maternal HFD exposure is associated with addiction-like behaviors later in life, potentially due to an attenuated dopaminergic reward system that increases susceptibility to compulsive behaviors [23,76,85,86,87,88,89]. Given this, one could speculate that the enhanced performance in the visual cue task suggests that reward-predictive cues are more salient for these offspring than for controls. One possible explanation for this phenomenon is that maternal HFD exposure alters the development of the mesolimbic dopamine system, which plays a crucial role in reward-based learning and motivation. Offspring of HFD-fed dams have been reported to exhibit increased dopaminergic activity in the nucleus accumbens and ventral tegmental area (VTA) [23,50,87,89,90]. Specifically, dopamine D2 receptor dysregulation and increased striatal dopamine turnover have been observed in maternal HFD models, leading to heightened responsiveness to reward-related cues [86,91]. This suggests that maternal HFD exposure enhances reinforcement-driven learning but does not necessarily improve general cognitive function, particularly in hippocampal-dependent tasks requiring spatial memory and executive control. The heightened sensitivity to reward-related cues may also be driven by an imbalance between dopaminergic and cholinergic signaling in the striatum, which has been implicated in compulsive behavior and impaired cognitive flexibility in maternal HFD models [92]. This aligns with human neuroimaging studies showing that individuals with obesity exhibit hyperresponsiveness to food cues and reward stimuli, similar to addiction patterns [93]. Furthermore, studies have demonstrated opioid system dysregulation in offspring of HFD-fed dams, which could further amplify reward-driven behaviors [89,94]. Given that the opioid system modulates reward salience and motivation, these neurobiological alterations may explain why HFD-exposed offspring excel in visually guided tasks that prioritize reward expectancy and reinforcement learning but still exhibit deficits in more cognitively demanding spatial navigation tasks [95]. These findings suggest that while maternal HFD exposure leads to broad cognitive impairments, it may simultaneously enhance reward-related learning, which could have important implications for behavior. Future studies should further investigate whether targeted interventions, such as dopamine-modulating treatments or early cognitive training, could mitigate the cognitive deficits while preserving adaptive reward-based learning mechanisms.

Future research should explore the efficacy of targeted interventions in reversing maternal HFD-induced cognitive deficits. Studies indicate that interventions such as maternal dietary modifications during gestation and lactation, as well as postnatal cognitive training, could mitigate the risk of long-term cognitive impairments in offspring [96,97,98]. For example, postnatal administration of choline, an essential nutrient for brain development, has been shown to improve memory performance in offspring exposed to prenatal insults [99,100]. However, some reports suggest that the effectiveness of these interventions may depend on the timing and severity of maternal HFD exposure, with late-stage interventions being less effective than those implemented early in development [43]. Addressing these questions could inform future public health initiatives aimed at reducing neurodevelopmental risks associated with maternal overnutrition.

## 5. Conclusions

In summary, our findings provide evidence that maternal overnutrition disrupts fetal brain development, leading to long-term cognitive impairments. The differences observed between spatial novelty preference (Y-maze) and spatial matching-to-position tasks suggest that distinct neurobiological mechanisms contribute to these deficits. Further studies are needed to elucidate the specific contributions of NMDA receptor dysfunction, synaptic plasticity alterations, and stress-related mechanisms in mediating the observed cognitive deficits. With obesity rates increasing among women of reproductive age, these findings emphasize the potential intergenerational effects of maternal nutrition on cognitive health and highlight the importance of targeted interventions to reduce these risks.

## Data Availability

The original contributions presented in the study are included in the article, further inquiries can be directed to the corresponding author.
